# Characterization of a Novel Population of Low-Density Granulocytes Associated with Disease Severity in HIV-1 Infection

**DOI:** 10.1371/journal.pone.0048939

**Published:** 2012-11-13

**Authors:** Thomas Cloke, Markus Munder, Graham Taylor, Ingrid Müller, Pascale Kropf

**Affiliations:** 1 Department of Medicine, Section of Immunology, Faculty of Medicine, Imperial College London, London, United Kingdom; 2 Third Department of Medicine (Hematology, Oncology, and Pneumology), University Medical Center Mainz, Mainz, Germany; 3 Section of Infectious Diseases, Department of Medicine, Imperial College London, London, United Kingdom; 4 Immunology and Infection Department, London School of Hygiene and Tropical Medicine, London, United Kingdom; Imperial College London, United Kingdom

## Abstract

The mechanisms resulting in progressive immune dysfunction during the chronic phase of HIV infection are not fully understood. We have previously shown that arginase, an enzyme with potent immunosuppressive properties, is increased in HIV seropositive (HIV+) patients with low CD4^+^ T cell counts. Here we show that the cells expressing arginase in peripheral blood mononuclear cells of HIV+ patients are low-density granulocytes (LDGs) and that whereas these cells have a similar morphology to normal-density granulocyte, they are phenotypically different. Importantly, our results reveal that increased frequencies of LDGs correlate with disease severity in HIV+ patients.

## Introduction

Since the initial report of AIDS in 1981, approximately 60 million people have become infected with HIV of which more than 30 million have died. Although depletion of CD4^+^ T cells explains much of the immune suppression in HIV infected individuals, the precise reasons for the onset of immunopathology during HIV infection are not yet fully understood [1], [2], [3], [4].

Arginase, an enzyme of the urea cycle, can also be expressed in cells of the immune system and has been shown to exert potent immunoregulatory functions: a reduction in the bioavailability of L-arginine by arginase results in impaired T cell responses, characterized by down-regulation of T cell proliferation, reduced expression of CD3ζ and cytokine production [5], [6], [7], [8]. To date, two mechanisms by which arginase depletes L-arginine have been described:

1. Transport of extracellular L-arginine into the cells by cationic amino acid transporter (CAT)2B to make it accessible for catabolization by arginase results in depletion of L-arginine in the microenvironment [9], [10];
2. Arginase can be released by neutrophils into the extracellular milieu, where it binds L-arginine and thereby reduces the level of free L-arginine available to T cells [11].

We have previously shown that arginase activity is higher in peripheral blood mononuclear cells (PBMCs) isolated from HIV-1 infected individuals with low CD4^+^ T cell counts and that this coincided with lower levels of L-arginine [12]. In the present study, we characterized the phenotype of arginase-expressing cells in HIV+ patients and tested the hypothesis that their frequency correlated with markers of disease severity.

## Materials and Methods

### Subjects and Samples

Thirtytwo HIV seropositive (HIV+) treatment-naïve individuals (mean age 42.1±12.2 years) were recruited from St Mary’s Hospital and 11 healthy volunteers (mean age 35±4.7 years) were recruited as control subjects. The study was approved by the National Research Ethics Service (05/Q0410/93) and all individuals gave written, informed consent before participation.

Twenty ml of peripheral blood was collected in EDTA tubes and PBMCs were isolated by density gradient centrifugation on Histopaque^®^-1077 (Sigma). Neutrophils were isolated from the erythrocyte fraction by dextran sulphate sedimentation [13].

#### Flow cytometry

The following antibodies were used: CD14^FITC^, CD15^PE^ (BD Pharmingen), Arginase1^Alexa Fluor® 647^ (Hycult Biotechnology), CD11b^PerCP-eFluro710^, CD16^eFluro450^, CD33^PE-Cy7^ (eBioscience), CD13^APC-Cy7^ (Biolegend), CD66b^FITC^ and CD63^FITC^ (Beckman Coulter) (Tables 1 and 2). Analysis was performed on an FACS Canto II (BD Bioscience) and results were analyzed using FlowJo v8.7 (Tree Star, Ashland, OR).

**Table 1:** Phenotype Panel.

Antigen	Fluorophore	Clone	**Isotype**	**Produced by**	**Product code**	**Volume used (µL)**	**Quantity (µg)**
CD15	PE	H198	mouse IgM	BD Pharmingen	555402	20	NP
CD11b	PerCP-eFluor710	ICRF44	mouse IgG1	eBioscience	46–0118	3	0.075
CD13	APC-Cy7	WM15	mouse IgG1	Biolegend	301710	7	NP
CD16	eFluor 450	eBioCB16	mouse IgG1	eBioscience	48–0168	3	0.15
CD33	PE-Cy7	WM-53	mouse IgG1	eBioscience	25–0338	3	0.15
CD66b	FITC	80H3	mouse IgG1	Beckman Coulter	IM05310	3	NP

LDGs and NDGs were isolated as described in materials and methods and the expression levels of phenotypic markers were determined by flow cytometry.

NP  =  Not provided by manufacturer.

**Table 2:** ARGINASE 1 Panel.

**Antigen**	**Fluorophore**	**Clone**	**Isotype**	**Produced by**	**Product code**	**Volume used (µL)**	**Quantity (µg)**
CD15	PE	H198	mouse IgM	BD Pharmingen	555402	20	NP
CD13	APC-Cy7	WM15	mouse IgG1	Biolegend	301710	7	NP
CD16	eFluor 450	eBioCB16	mouse IgG1	eBioscience	48–0168	3	0.15
CD63	FITC	CLBGran/12	mouse IgG1	Beckman Coulter	IM1165U	14	NP
Arginase 1	Alexa Fluor® 647	6G3	mouse IgG1	Hycult biotech.	HM2162	2.75	0.275

LDGs and NDGs were isolated as described in materials and methods and the expression levels of phenotypic markers were determined by flow cytometry.

NP  =  Not provided by manufacturer.

#### Isolation of CD15^+^ cells

CD3^+^ and CD14^+^ cells were removed by positive selection, CD15^+^ cells were incubated with anti-CD15^PE^ and selected using EasySep PE Positive Selection Kit (EasySep, Stem Cell Technologies, France). Purity of CD15^+^ cells was checked by flow cytometry (>95%). The remaining cells were transferred onto a microscope slide (Thermo Scientific, United Kingdom) using a cytospin centrifuge and stained with hematoxylin and eosin (H&E) (Reagena, Gamidor, United Kingdom).

**Figure 1:**
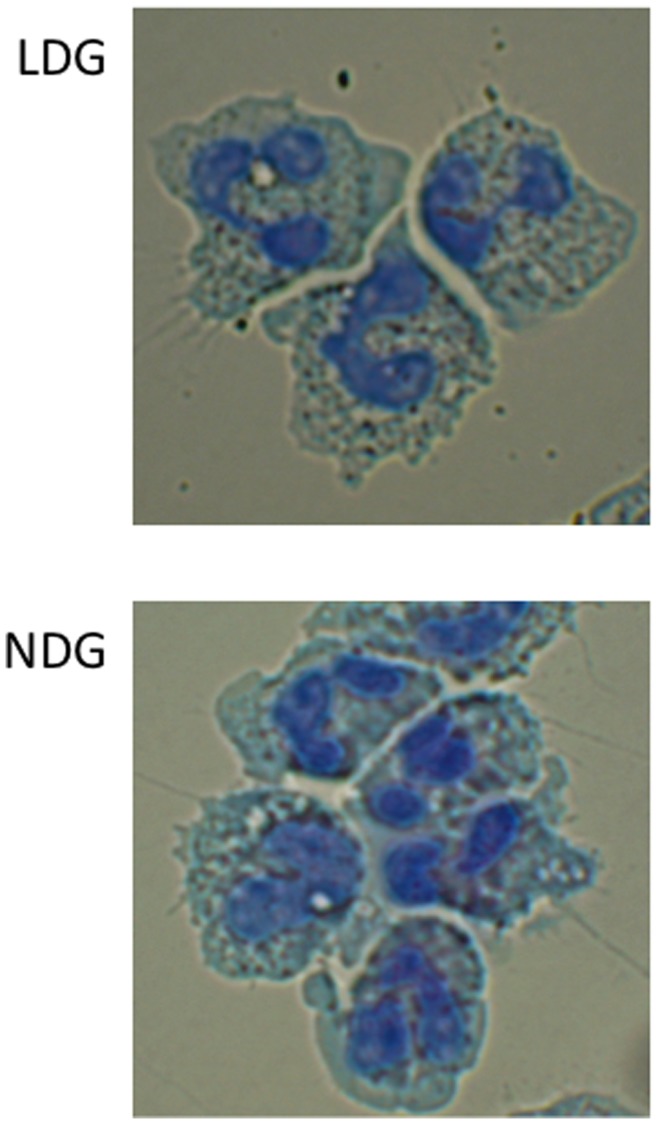
Morphology of LDGs and NDGs. LDGs and NDGs were isolated as described in materials and methods and their morphology was compared after H&E staining. Data show the results of one representative experiment out of five independent experiments.

**Figure 2:**
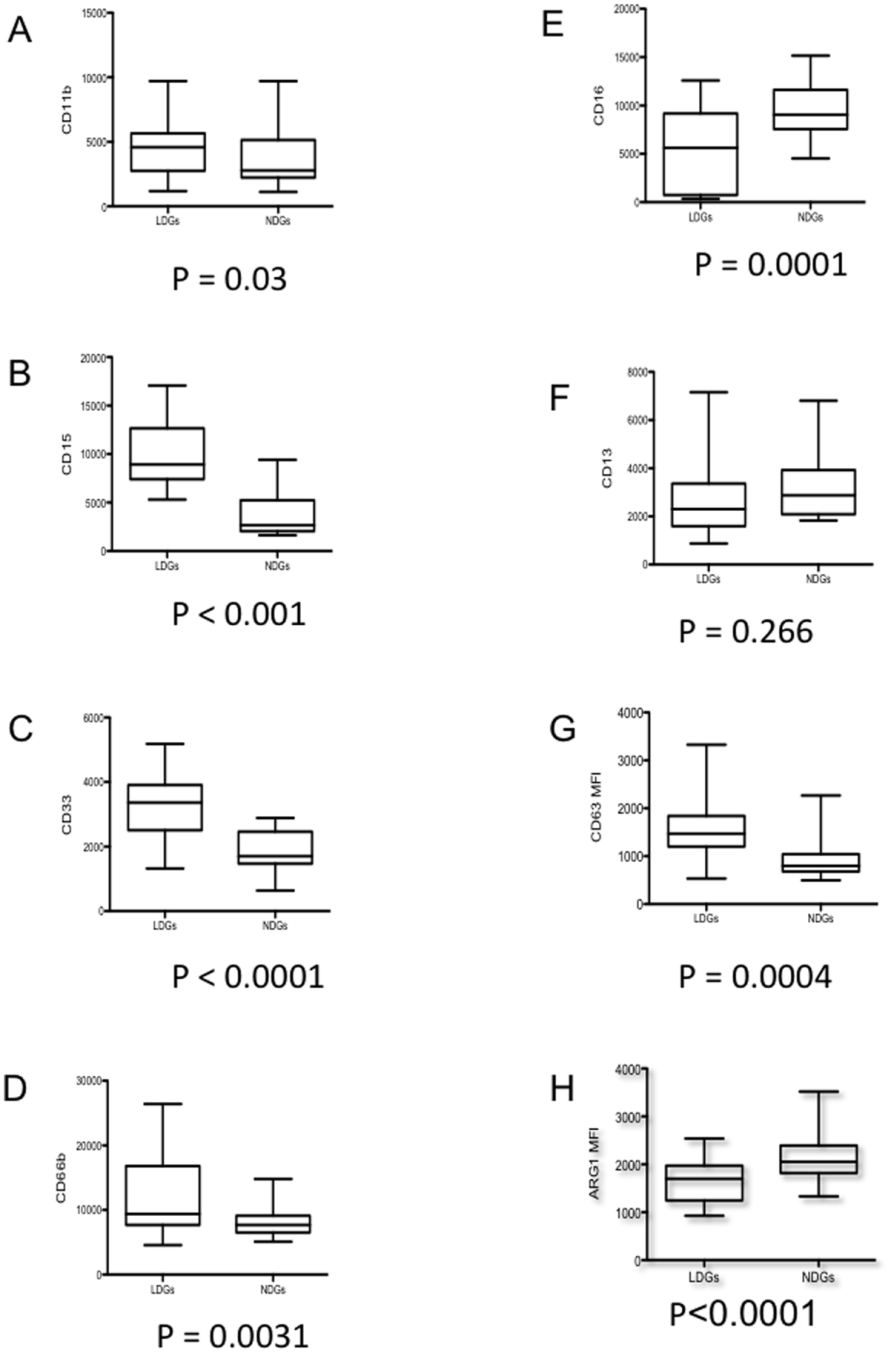
Phenotypic analysis of LDGs and NDGs. LDGs and NDGs were isolated as described in materials and methods (n = 22) and the expression levels of CD11b (A), CD15 (B), CD33 (C), CD66b (D), CD16 (E), CD13 (F), CD63 (G) and arginase 1 (H) were determined by flow cytometry. Isotype controls: <1%. Statistical significance was determined by a two-tailed Mann-Whitney test. Box = interquartile range and median; whiskers = range.

### Statistical Analyses

Data were evaluated for statistical differences using a Wilcoxon signed rank test, a two-tailed Mann-Whitney test and a Spearman’s rank test when appropriate (GraphPad Prism 5); differences were considered statistically significant at *p*<0.05. Unless otherwise specified, results are expressed as median±SEM.

**Table 3:** MFI of phenotypic markers of LDGs and NDGs.

	Controls		Controls	HIV+ patients
Phenotypic markers	LDGs (MFI)	NDGs (MFI)	% change	% change
CD11b	3267±587	4334±183	−6.9±10.4	29.0±9.2%
CD13	305±200	2196±167	−65.4±11.0	−7.5±10.0%
CD15	5313±270	2267±201	106.1±12.2	239.4±46.6%
CD16	384±81	15678±1197	−97.2±0.4	−46.4±8.4%
CD33	1385±327	506±95	176.4±19.1	82.8±12.1%
CD66b	11954±929	8393±326	58.0±9.7	61.2±17.3
CD63	2077±129	987±68	111.8±18.7	84.7±19.3
Arginase 1	11899±592	16463±884	−29.5±5.1	−22.8±4.5

LDGs and NDGs were isolated as described in materials and methods and the expression levels of phenotypic markers were determined by flow cytometry (median±SEM). The percentage increase or decrease in MFI was calculated for controls (n = 11) and HIV+ patients (figures 2A–G, n = 22).

## Results

### Phenotypic Characterization of Different Populations of Granulocytes

We have previously shown that arginase activity is significantly higher in PBMCs of HIV+ individuals with low CD4^+^ T cell counts. Furthermore, we showed that the cells expressing arginase in the PBMC fraction are granulocytes [12]. Typically granulocytes sediment with erythrocytes following density gradient centrifugation. Based on this difference in density we refer to granulocytes that co-purify in the PBMC fraction as low-density granulocytes (LDGs) and granulocytes which sediment with erythrocytes as normal-densitiy granulocytes (NDGs). To determine whether these two populations of granulocytes can be distinguished morphologically and phenotypically, we purified LDGs and NDGs and stained both populations with H&E. As shown in Figure 1, despite a difference in density, these two populations are morphologically similar: they are mature, segmented neutrophils. To answer whether they differ phenotypically, we assessed the expression levels of a panel of phenotypic markers of neutrophils. The mean fluorescence intensities (MFI) of CD11b, CD15, CD33 and CD66b were significantly greater (Figures 2 A–D) on LDGs than on NDGs. CD16 MFI was significantly reduced on LDGs (Figure 2 E). No significant difference was observed in the MFI of CD13 (Figure 2 F).

To establish whether this phenotype differs from that of healthy individuals, we measured the expression levels of the same panel of cell surface markers on LDGs and NDGs from healthy individuals. As shown in table 3, there was also an increase in the MFI of CD15, CD33, CD63 and CD66b, and a decrease in the MFI of CD16 and arginase 1. There were no significant differences in the MFI of CD11b. However, there was a significant decrease in CD13 MFI on LDGs as compared to NDGs (Table 3).

The blood from the controls and HIV+ patient with high or low CD4^+^ T cell counts was processed immediately and in the exact same way, therefore excluding that any differences observed were due to the handling procedure.

These results show that NDGs and LDGs are morphologically mature neutrophils, but that they differ in the expression level of markers of polymorphonuclear cells.

### LDGs Express Higher Levels of CD63 and Lower Levels of Arginase

In neutrophils, arginase is localized in azurophilic granules [13], which express CD63; following activation, neutrophils degranulate and the release of arginase-containing azurophilic granules results in the incorporation of CD63 into the cell surface membrane of neutrophils [14], [15], [16], [17]. The arginase released by activated neutrophils has been shown to induce a profound suppression of T cell proliferation and cytokine synthesis [18]. To test whether LDGs have released their azurophilic granules, we measured the MFI of CD63 on LDGs and the MFI of arginase 1 in LDGs. As shown in Figures 2 G–H, CD63 MFI was significantly higher (*p*<0.0001) and arginase MFI was significantly lower (*p* = 0.0004) in LDGs as compared to NDGs. These results suggest that LDGs have degranulated and released their arginase.

### The Frequency of LDGs Correlates with Markers of Disease Severity

**Figure 3:**
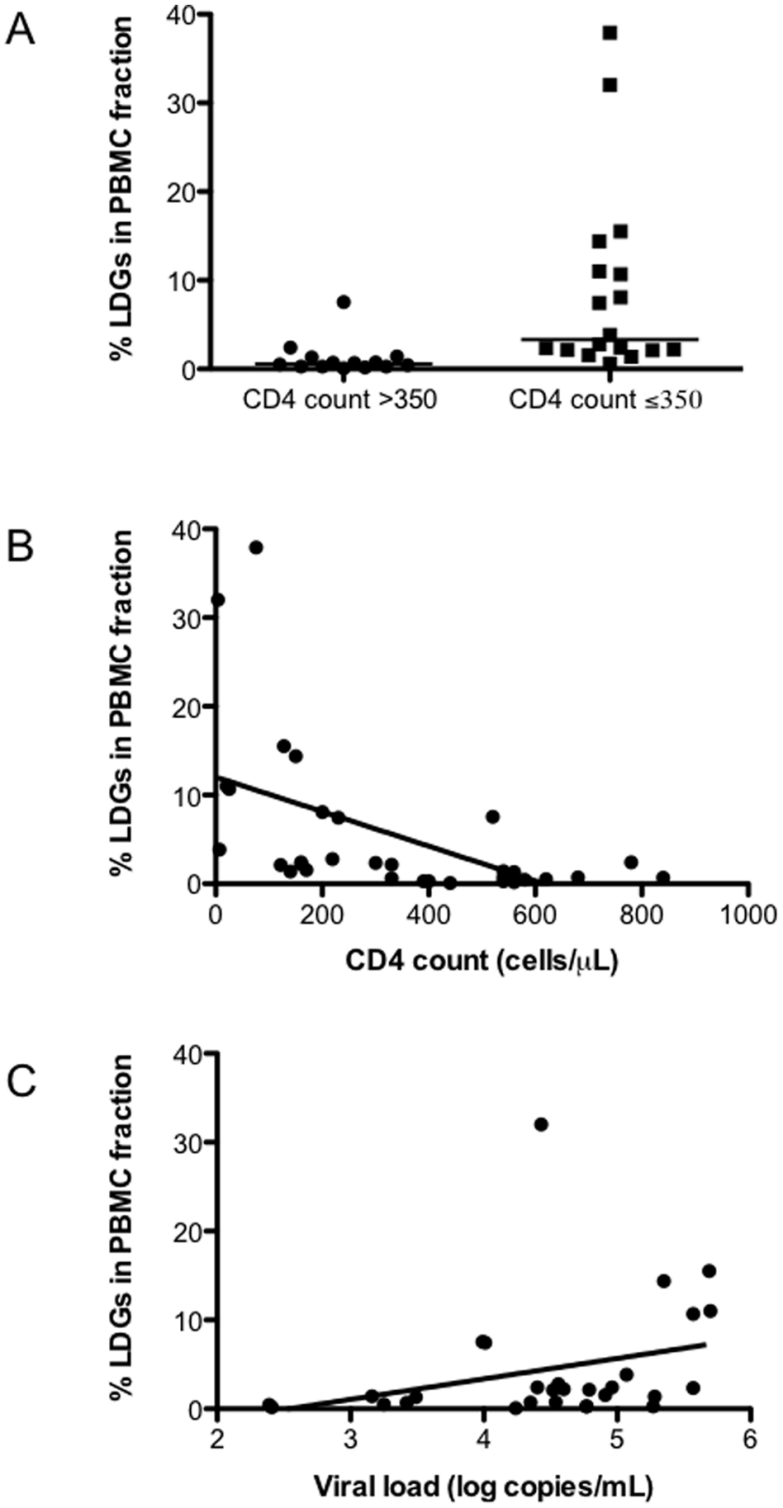
Frequency of LDGs in PBMCs of HIV patients. PBMCs from HIV+ patients with CD4^+^ T cell counts >350 (n = 14) or <350 cells/µL (n = 18) were isolated by Ficoll gradient and the frequency of CD15^+^ arginase^+^ cells was determined by flow cytometry, statistical significance was determined by a two-tailed Mann-Whitney test (A). Correlation between %LDGs and CD4^+^ T cell counts (B) or viral load (C), statistical significance was determined by a Spearman’s rank test. Isotype controls: <1%.

Markers of disease severity in HIV-1 infection include CD4+ T cell count and viral load. Consequently we assessed whether the frequency of LDGs in healthy controls and HIV patients correlated with markers of disease severity. First, we compared the frequency of LDGs in healthy controls and HIV patients. The frequency of LDG was significantly lower in healthy controls as compared to HIV+ patients with CD4^+^ T cell counts >350 cells/µl (0.24±0.3 vs 0.60±0.52, *p* = 0.0160) and HIV+ patients with CD4^+^ T cell counts <350 cells/µl (0.24±0.3 vs 3.33±2.51, *p*<0.0001) (data not illustrated). Next we determined whether the frequencies of LDGs varied between individuals with low or high CD4^+^ T cell counts. As shown in Figure 3A, the percentage of LDGs in the PBMCs of HIV+ individuals with CD4^+^ T cell counts <350 cells/µl was significantly higher (3.33±2.51 vs 0.60±0.52, *p* = 0.0001). Similar results were obtained with the absolute counts of LDGs per ml of blood (6.17±6.78×10^4^ vs 1.94±1.41×10^4^, *p* = 0.0037). To assess the potential clinical significance of this difference, we tested for a correlation between the percentages of LDGs in the PBMCs against absolute CD4^+^ T cell count and HIV-1 viral load. Results in Figure 3B show a strong inverse correlation between CD4^+^ T cell counts and percentage of LDGs (r = −0.6908, p<0.0001). There was also a significant correlation between HIV-1 viral load and percentage of LDGs (Figure 3C, r = 0.4661, *p* = 0.0108). These results show that in HIV+ patients, the frequency of LDGs present in PBMCs correlated with markers of disease severity.

## Discussion

Arginase-induced L-arginine catabolism is a well-established mechanism of immune suppression [5], [6], [7], [8]. We have recently shown that arginase activity is abnormally high in PBMCs of untreated HIV+ individuals with low CD4^+^ T cell counts and that this coincides with lower levels of L-arginine [12]. Here we consolidate and extend our previous results and show that the frequency of arginase-expressing cells is closely associated with the two most widely accepted markers of disease severity: CD4^+^ T cell counts and plasma HIV-1 viral loads.

Following density gradient centrifugation, LDGs co-purify in the PBMC fraction, whereas NDGs sediment with erythrocytes. This difference could result from i) different degrees of maturation and ii) different activation and degranulation states:

- During neutrophil maturation there is a change in expression of surface antigens [19]. In untreated HIV+ individuals, LDGs display high expression levels of CD33, a marker of immature neutrophils, and lower levels of CD16, a marker found on mature neutrophils, suggesting that these cells are immature [19]. A study by Brandau *et al.* identified immature granulocytes from individuals with cancer that co-purify in the PBMC fraction [20]. However, our results show that LDGs are morphologically similar to NDGs, as they are segmented neutrophils. We cannot exclude the possibility that the population of LDGs isolated from HIV+ individuals represents a heterogeneous population of neutrophils that does not exclusively comprise mature activated cells; indeed, Rodriguez et al showed that in renal cell carcinoma, <10% of the myeloid derived suppressor cells (MDSCs) were immature granulocytes and that ∼90% were mature segmented granulocytes [21].
- The degree of activation of neutrophils and degranulation depends on the strength of the activating signal: the order of granule release follows a strict hierarchy requiring increasing activation: 1) secretory granules; 2) gelatinous (tertiary) granules; 3) specific (secondary) granules and 4) azurophilic (primary) granules. From the panel of markers used in the present study, the following markers were significantly increased on LDGs: CD11b, present in the membrane of secretory vesicles, gelatinase granules and specific granules; CD63, found in the membrane of azurophilic granules; and CD66b, detected in the membrane of specific granules. Further, the intensity of arginase, which is present in azurophilic granules [13], was lower in LDGs as compared to NDGs. Our results suggest that LDGs are activated neutrophils that have degranulated because they have increased cell surface expression of CD66b, CD63 and CD11b and decreased intracellular arginase 1 expression.

The presence of CD15^+^ granulocytes in the PBMC fraction has been demonstrated in a number of different conditions including cancer, pregnancy, trauma and SLE, each of which is frequently accompanied by a degree of immune suppression. We have shown that arginase-induced L-arginine depletion and the subsequent T cell inhibition is a mechanism of immune suppression that is restricted to the site of pathology [22]. Therefore, it is possible that the increased frequencies of LDGs we observed in the peripheral blood of HIV+ individuals are only a weak reflection of the events occurring at the principal sites of HIV infection, i.e. in the solid lymphoid tissue. Indeed, a recent study showed in a model of prostate-specific inflammation, that MDSCs isolated from the site of inflammation were more immunosuppressive than those isolated from the periphery [23].

The impact of arginase-induced L-arginine depletion on T cell effector functions has been well established [5], [6], [7], [8]. In HIV+ individuals with low CD4^+^ T cell counts, we and others have already reported a pronounced downregulation of CD3ζ, which is one of the hallmarks of T cell suppression induced by L-arginine depletion [12]. We propose that the following mechanism contributes to poor T cell function in these individuals: LDGs release arginase, which reduces the concentration of free L-arginine in the microenvironment [12], thereby preventing efficient T cell responses. Indeed, we have previously shown that PMN release their arginase and that this inhibits T cell proliferation in an arginase-dependent manner [18].

More work is needed to understand the causes of the observed neutrophil activation and the subsequent release of arginase, as it is not yet not possible to answer whether it is the virus or the immune system or a combination of both that accounts for the increased frequency of activated low-density granulocytes in HIV+ patients. A better understanding of the complex mechanisms leading to progressively impaired immune functions might prove helpful in improving the existing treatment not only for HIV+ individuals, but also for other chronic infectious diseases such as tuberculosis and leishmaniasis, in which a degree of immune suppression may be caused by abnormal arginase activity.
